# Supplementary material for: Novel Copper(II) Complexes with Dipinodiazafluorene Ligands: Synthesis, Structure, Magnetic and Catalytic Properties
Source: Molecules. 2022 Jun 24;27(13):4072. doi: 10.3390/molecules27134072 (PMC9268630; doi:10.3390/molecules27134072)
Supplement: Supplementary file 1 [file molecules-27-04072-s001.zip › molecules-1784896-supplementary.pdf]

Novel Copper(II) Complexes with Dipinodiazafluorene Ligands: Synthesis, Structure, Magnetic and Catalytic Properties

Iakov S. Fomenko [1], Medhanie Afewerki [2], Marko I. Gongola [2], Eugene S. Vasilyev [3], Lidia S. Shul'pina [4],
Nikolay S. Ikonnikov [4], Georgiy B. Shul'pin [5,6], Denis G. Samsonenko [1], Vadim V. Yanshole [7], Vladimir A. Nadolinny [1], Alexander N. Lavrov [1], Alexey V. Tkachev [3] and Artem L. Gushchin [1,*]

[1] Nikolaev Institute of Inorganic Chemistry, Siberian Branch of Russian Academy of Sciences, 3 Acad. Lavrentiev Ave., Novosibirsk 630090, Russia; fom1-93@mail.ru (I.S.F.); denis@niic.nsc.ru (D.G.S.); spectr@niic.nsc.ru (V.A.N.); lavrov@niic.nsc.ru (A.N.L.)
[2] Department of natural sciences Novosibirsk State University, 1 Pirogova Str., Novosibirsk 630090, Russia; medahaben@gmail.com (M.A.); m.gongola@g.nsu.ru (M.I.G.)
[3] Vorozhtsov Novosibirsk Institute of Organic Chemistry, Siberian Branch of Russian Academy of Sciences, 9 Acad. Lavrentiev Ave., Novosibirsk 630090, Russia; vasilyev@nioch.nsc.ru (E.S.V.);
atkachev@nioch.nsc.ru (A.V.T.)
[4] A.N. Nesmeyanov Institute of Organoelement Compounds, Russian Academy of Sciences, ul. Vavilova. 28, Moscow 119991, Russia; shulpina@ineos.ac.ru (L.S.S.); ikonns@ineos.ac.ru (N.S.I.)
[5] N.N. Semenov Federal Research Center for Chemical Physics, Russian Academy of Sciences, ul. Kosygina, 4, Moscow 119991, Russia; gbsh@mail.ru
[6] Chemistry and Physics, Plekhanov Russian University of Economics, Stremyannyi Pereulok, 36, Moscow 117997, Russia
[7] International Tomography Center, Siberian Branch of Russian Academy of Sciences, 3a Institutskaya Str., Novosibirsk 630090, Russia; vadim.yanshole@tomo.nsc.ru
* Correspondence: gushchin@niic.nsc.ru

Table of contents

Figure S1. Mass spectrum of **1** in CH_3CN

Figure S2. Mass spectrum of **2** in CH_3CN

Figure S3. Mass spectrum of **3** in CH_3CN

Figure S4. Mass spectrum of **4** in CH_3CN

Table S1. Identified signals in the mass spectra of complexes **1-4** in acetonitrile

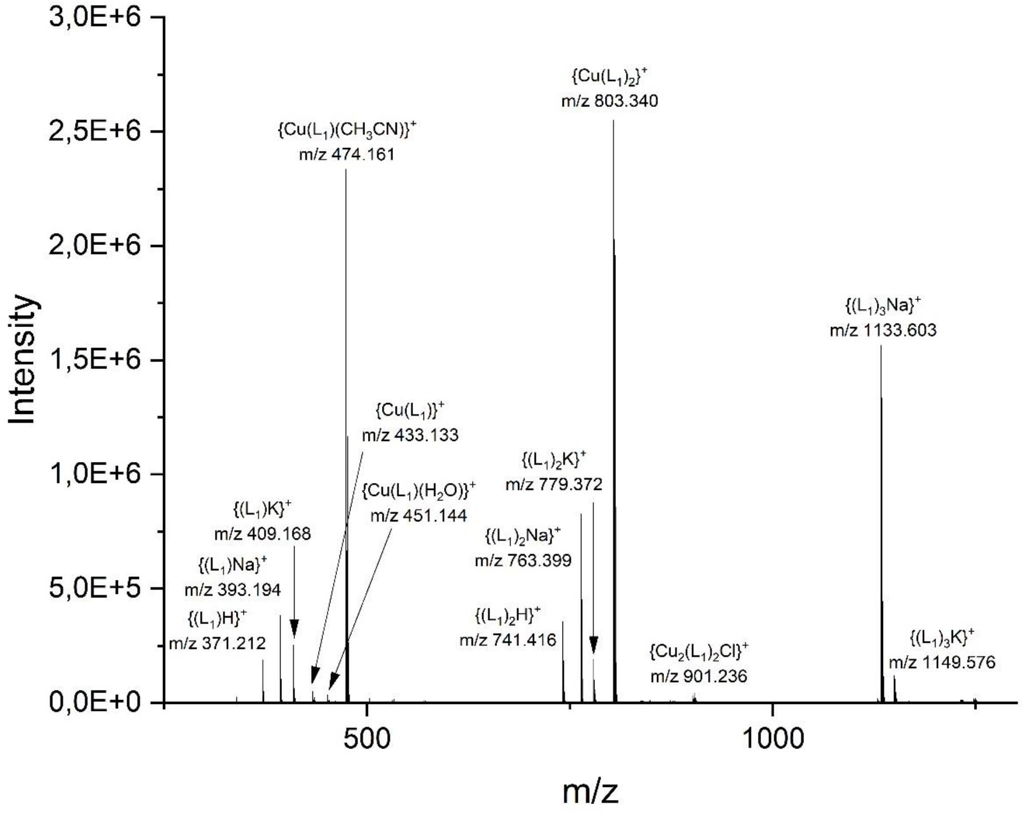

Figure S1. Mass spectrum of **1** in CH3CN

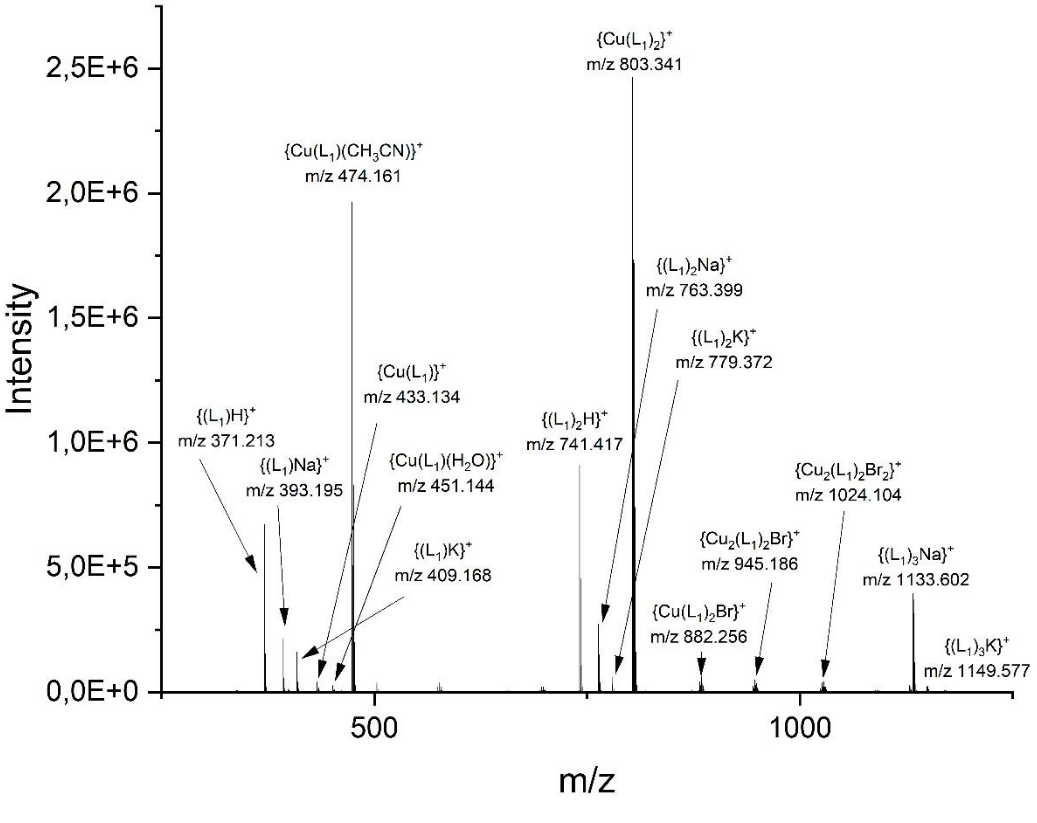

Figure S2. Mass spectrum of **2** in CH3CN

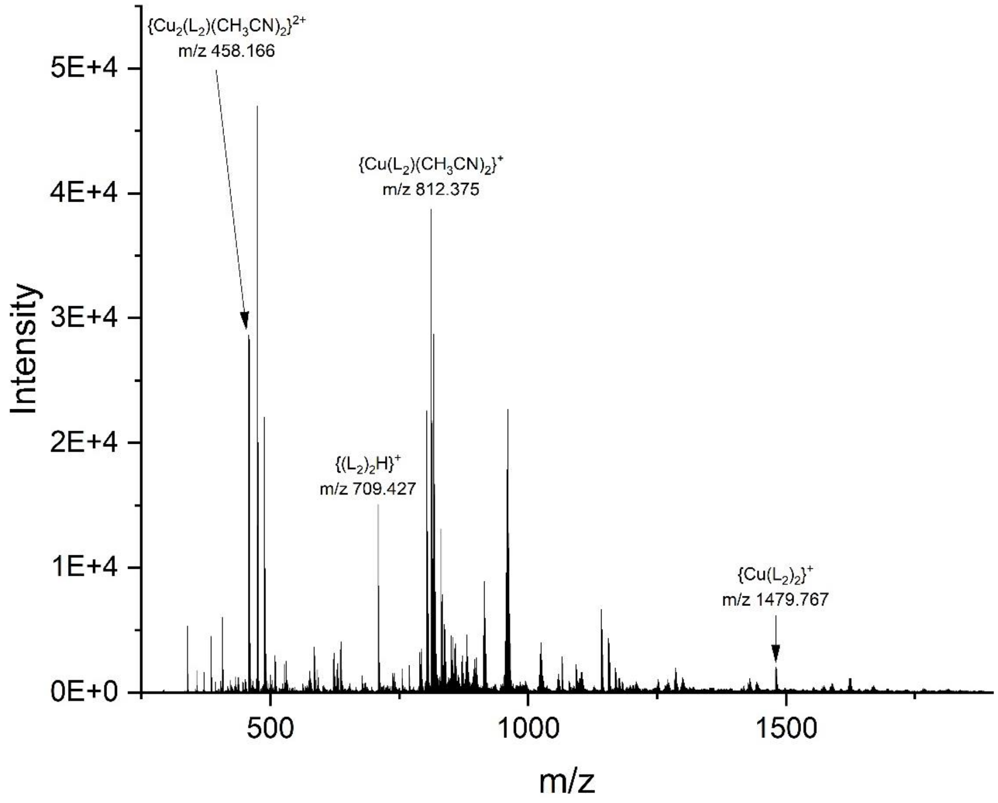

Figure S3. Mass spectrum of **3** in CH_3CN

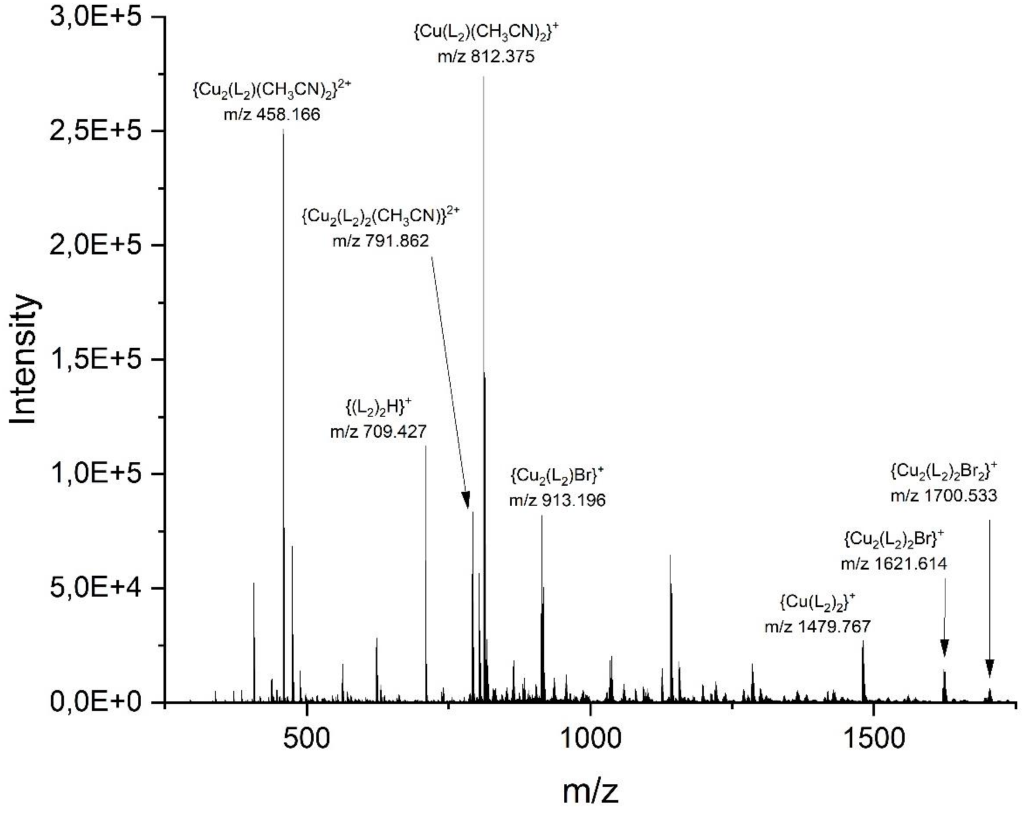

Figure S4. Mass spectrum of **4** in CH_3CN

Table S1. Identified signals in the mass spectra of complexes **1-4** in acetonitrile

**$[CuCl_2L_1]_2$ (1)**			
**Identified chemical formula**	**Measured m/z**	**Calculated m/z**	**Fragment**
$C_{25}H_{26}N_2O$ + H	371.212	371.212	L_1 + H
$C_{25}H_{26}N_2O$ + Na	393.194	393.194	L_1 + Na
$C_{25}H_{26}N_2O$ + K	409.168	409.168	L_1 + K
$C_{25}H_{26}N_2O$ + Cu	433.133	433.134	L_1 + Cu
$C_{25}H_{26}N_2O$ + Cu + H_2O	451.144	451.145	$Cu(L_1)(H_2O)$
$C_{25}H_{26}N_2O$+ Cu + CH_3CN	474.161	474.161	$Cu(L_1)(CH_3CN)$
$(C_{25}H_{26}N_2O)_2$ + H	741.416	741.417	$(L_1)_2$ + H
$(C_{25}H_{26}N_2O)_2$ + Na	763.399	763.399	$(L_1)_2$ + Na
$(C_{25}H_{26}N_2O)_2$ + K	779.372	779.373	$(L_1)_2$ + K
$(C_{25}H_{26}N_2O)_2$ + Cu	803.340	803.339	$(L_1)_2$ +Cu
$C_{50}H_{52}Cu_2N_4O_2Cl$	901.236	901.237	$Cu_2(L_1)_2Cl$
$C_{50}H_{52}Cu_2N_4O_2Cl_2$	936.205	936.206	$Cu_2(L_1)_2Cl_2$
$C_{50}H_{52}Cu_3N_4O_2Cl_2$	999.133	999.136	$Cu_3(L_1)_2Cl_2$
$(C_{25}H_{26}N_2O)_3$ + Na	1133.603	1133.603	$(L_1)_3$ + Na
$(C_{25}H_{26}N_2O)_3$ + K	1149.576	1149.577	$(L_1)_3$ + K
**$[CuBr_2L_1]_2$ (2)**			
$C_{25}H_{26}N_2O$ + H	371.213	371.212	L_1 + H
$C_{25}H_{26}N_2O$ + Na	393.195	393.194	L_1 + Na

$C_{25}H_{26}N_2O$ + K	409.168	409.168	L_1 + K
$C_{25}H_{26}N_2O$ + Cu	433.134	433.134	L_1 + Cu
$C_{25}H_{26}N_2O$ + Cu + H_2O	451.144	451.145	$Cu(L_1)(H_2O)$
$C_{25}H_{26}N_2O$ + Cu + CH_3CN	474.161	474.161	$Cu(L_1)(CH_3CN)$
$C_{25}H_{26}N_2OCuBr$	512.052	512.052	$Cu(L_1)Br$
$C_{25}H_{26}N_2OCu_2Br$	574.981	574.981	$Cu_2(L_1)Br$
$C_{25}H_{26}N_2OCu_2Br_2$	653.900	653.900	$Cu_2(L_1)Br_2$
$C_{25}H_{26}N_2OCu_2Br_2$ + CH_3CN	694.926	694.926	$Cu_2(L_1)Br_2(CH_3CN)$
$(C_{25}H_{26}N_2O)_2$ + H	741.417	741.417	$(L_1)_2$ + H
$(C_{25}H_{26}N_2O)_2$ + Na	763.399	763.399	$(L_1)_2$ + Na
$(C_{25}H_{26}N_2O)_2$ + K	779.372	779.373	$(L_1)_2$ + K
$(C_{25}H_{26}N_2O)_2$ + Cu	803.341	803.339	$(L_1)_2Cu$
$C_{50}H_{52}CuN_4O_2Br$	882.256	882.257	$Cu(L_1)_2Br$
$C_{50}H_{52}N_4O_2Cu_2Br$	945.186	945.187	$Cu_2(L_1)_2Br$
$C_{50}H_{52}N_4O_2Cu_2Br_2$	1024.104	1024.105	$Cu_2(L_1)_2Br_2$
$C_{50}H_{52}N_4O_2Cu_3Br_2$	1087.034	1087.034	$Cu_3(L_1)_2Br_2$
$(C_{25}H_{26}N_2O)_3$ + Na	1133.602	1133.603	$(L_1)_3$ + Na
$(C_{25}H_{26}N_2O)_3$ + K	1149.577	1149.577	$(L_1)_3$ + K
$C_{50}H_{52}N_4O_2Cu_3Br_3$	1165.952	1165.952	$Cu_3(L_1)_2Br_3$
**$[(CuCl_2)_2L_2]_n$ (3)**			
$(C_{50}H_{52}N_4)Cu_2(CH_3CN)_2^{2+}$	458.166	458.166	$Cu_2(L_2)(CH_3CN)_2$
$C_{50}H_{52}N_4$ + H	709.427	709.427	L_2 + H

$C_{50}H_{52}N_4$ + Na	731.408	731.409	L_2 + Na
$C_{50}H_{52}N_4Cu(CH_3CN)$	812.375	812.375	$Cu(L_2)(CH_3CN)$
$(C_{50}H_{52}N_4)_2Cu$	1479.767	1479.767	$Cu(L_2)_2$
**$[(CuBr_2)_2L_2]_n$ (4)**			
$(C_{50}H_{52}N_4)Cu_2(CH_3CN)_2^{2+}$	458.166	458.166	$Cu_2(L_2)(CH_3CN)_2$
$C_{50}H_{52}N_4$ + H	709.427	709.427	L_2+ H
$(C_{50}H_{52}N_4)_2Cu_2(CH_3CN)^{2+}$	791.862	791.862	$Cu_2(L_2)_2(CH_3CN)$
$C_{50}H_{52}N_4Cu(CH_3CN)$	812.375	812.375	$Cu(L_2)(CH_3CN)$
$(C_{50}H_{52}N_4)Cu_2Br$	913.196	913.197	$Cu_2(L_2)Br$
$(C_{50}H_{52}N_4)Cu_3Br_2$	1055.043	1055.044	$Cu_3(L_2)Br_2$
$(C_{50}H_{52}N_4)_2Cu$	1479.767	1479.767	$Cu(L_2)_2$
$(C_{50}H_{52}N_4)_2Cu_2Br$	1621.614	1621.615	$Cu_2(L_2)_2Br$
$(C_{50}H_{52}N_4)_2Cu_2Br_2$	1700.533	1700.534	$Cu_2(L_2)_2Br_2$
